# Long-Term Potentiation of Mossy Fiber Feedforward Inhibition of CA3 Pyramidal Cells Maintains E/I Balance in Epilepsy Model

**DOI:** 10.1523/ENEURO.0375-21.2021

**Published:** 2022-01-06

**Authors:** Enhui Pan, Ram S. Puranam, James O. McNamara

**Affiliations:** 1 Department of Neurology, Duke University School of Medicine, Durham, NC 27710; 2 Department of Neurobiology, Duke University School of Medicine, Durham, NC 27710; 3 Department of Pharmacology and Molecular Cancer Biology, Duke University School of Medicine, Durham, NC 27710

**Keywords:** CA3 pyramidal cells, feedforward inhibition, homeostatic, mossy fiber, status epilepticus

## Abstract

Insight into the cellular and circuit mechanisms underlying development of temporal lobe epilepsy (TLE) will provide a foundation for improved therapies. We studied a model in which an episode of prolonged seizures is followed by recovery lasting two weeks before emergence of spontaneous recurrent seizures. We focused on the interval between the prolonged seizures and the late onset recurrent seizures. We investigated the hippocampal mossy fiber CA3 pyramidal cell microcircuit in models spanning *in vitro*, *in vivo*, and *ex vivo* preparations. Expression of channelrhodopsin-2 in the dentate granule cells of *DGC ChR* mice enabled the selective activation of mossy fiber axons. *In vivo* studies revealed marked potentiation of mossy fiber evoked field potentials in hippocampal CA3 beginning within hours following seizures, a potentiation which persisted at least 7 d. Stimulation of mossy fibers in hippocampal slices *in vitro* using patterns of activity mimicking seizures induced LTP not only of the monosynaptic EPSC but also of the disynaptic IPSC of CA3 pyramidal cells. *Ex vivo* studies of slices isolated following seizures revealed evidence of LTP of mossy fiber evoked EPSC and disynaptic IPSC of CA3 pyramidal cells. We suggest that activation of dentate granule cells during seizures induces these plasticities *in vivo* and the retained balance of synaptic excitation and inhibition limits excessive activation of CA3 pyramidal cells, thereby protecting animals from spontaneous recurrent seizures at this interval following status epilepticus.

## Significance Statement

The hippocampal mossy fiber CA3 pyramidal cell circuit is essential for normal memory function. Malfunction of this circuit has been identified in diverse diseases including epilepsy. Excessive neuronal activity induces pathologic increases of excitatory communication between neurons in this circuit in an animal model of epilepsy, increases likely to predispose to epileptic seizures. Importantly, the major inhibitory input to the CA3 pyramidal cells was also strengthened. The retained balance of excitation and inhibition likely limits excessive activation of CA3 pyramidal cells and protects animals from seizures at this interval following the insult.

## Introduction

Temporal lobe epilepsy (TLE) is a common and commonly devastating disease. TLE can arise as a consequence of diverse insults, one of which is an episode of prolonged seizures or status epilepticus (Annegers et al., 1987; French et al., 1993; Tsai et al., 2009; Pitkänen and Lukasiuk, 2011; Hesdorffer et al., 2016). Understanding the synaptic and microcircuit mechanisms underlying this disorder will hopefully reveal novel strategies for developing preventive and/or disease modifying therapies. Availability of animal models in which experimental induction of status epilepticus causes TLE provides an opportunity to elucidate the underlying mechanisms.

The hippocampus is a pivotal structure in TLE, evidenced in part by reduction of seizures following hippocampal lesions in patients with medically refractory TLE (Sheikh et al., 2019). Analyses of experimental models reveal striking increases of excitability of the dentate gyrus during epileptogenesis and chronic epilepsy (Tauck and Nadler, 1985; Parent et al., 1997; Kobayashi and Buckmaster, 2003; Dengler et al., 2017), underscoring the rationale for examining the synaptic station of the trisynaptic network immediately downstream from the granule cells, the synaptic input to the microcircuitry of CA3. Giant boutons of granule cell mossy fiber axons synapse on thorny excrescent spines of CA3 pyramidal cells whereas filopodial extensions of these boutons synapse selectively on interneurons in CA3, the number of filopodial connections exceeding the giant boutons by at least tenfold (Acsády et al., 1998). These connections underlie mossy fiber evoked monosynaptic excitatory input and disynaptic feedforward inhibitory input to CA3 pyramidal cells (Mori et al., 2007; Torborg et al., 2010; Rebola et al., 2017; Vandael et al., 2020). Here, we demonstrate that status epilepticus induced persistent increases of mossy fiber evoked field potentials in CA3 *in vivo.* Subsequent *ex vivo* studies investigated the effects of status epilepticus on the mossy fiber evoked monosynaptic EPSC and disynaptic IPSC of CA3 pyramidal cells.

## Materials and Methods

Animals were handled according to the *National Institutes of Health* Guide for the Care and Use of the Laboratory Animals, and protocols were approved by Duke University Animal Care and Welfare Committee.

### Mice

Crossing transgenic mice with the *Dock 10* promoter driving expression of Cre recombinase (*Dock 10 cre*) to a floxed Channel Rhodopsin mouse (Rosa-CAG-LSL-ChR2(H134R)-EYFP-WPRE) generated progeny in which expression of Channel Rhodopsin in hippocampus is restricted to dentate granule cells [dentate granule cell Channel Rhodopsin (*DGC ChR*); Kohara et al., 2014]. The genotype of each animal was verified twice using PCR of genomic DNA isolated from the tail before and after experiments. The *Dock 10 Cre* line was generously provided by Susumu Tonegawa (Massachusetts Institute of Technology). The floxed Channel Rhodopsin line was obtained from The Jackson Laboratory (#024109). Wild-type C57/Bl6 mice were obtained from Charles River.

### Seizure model

Either vehicle (PBS) or kainic acid (KA; 16 mg/kg) was infused into tail vein of awake, gently restrained mice over a period ∼2 min. Infusion of KA induced repeated limbic and tonic clonic seizures (“status epilepticus”) beginning within a few minutes and persisting for ∼45–60 min before remitting spontaneously without requiring antiseizure drugs as reported elsewhere (Drysdale et al., 2021). Behavioral seizures were video monitored for at least 40 min and status epilepticus confirmed by offline review. Animals typically resumed normal behavior within 2–3 h. Examining the effects of varying doses of KA led to selection of 16 mg/kg, because it induced behavioral seizures with a mortality of 20% or less (Drysdale et al., 2021).

### Optogenetic activation of mossy fibers *in vivo*

A 200 μm diameter optical fiber with numerical aperture 0.39 (Thorlabs) was placed in the suprapyramidal blade of the dentate gyrus of the left dorsal hippocampus using stereotaxic coordinates [relative to bregma, anterior posterior (AP) −2.0, mediolateral (ML) −1.0, dorsoventral (DV) −1.8; in mm] under isoflurane anesthesia. A bipolar recording electrode was placed in left hippocampal CA3 region of GC/ChR mice using stereotaxic guidance (AP ±2.0, ML ±2.5, and DV –2.5 (in mm) and placement optimized by recording local field potentials evoked by optical stimulation (IKE-473-200 OP, Ikecool; wavelength 473 nm). The stereotaxic coordinates identified after optimization of the recording electrode placement in the left hippocampus were used to place a bipolar electrode in the right hippocampus. Light evoked responses were delivered to freely moving animals in pairs separated by 60 ms at a frequency of 0.033 Hz for a period of 10 min between 8 and 10 A.M. Modifying TTL pulse duration from 0.5 to 1.5 ms in 0.1-ms steps was used to produce an input output curve. Response amplitudes varying by no more than 20% in input output curves collected on consecutive days were deemed stable and pulse duration producing response 30% of maximum was used in subsequent experiments following treatment with either PBS or KA. The LFP signal was amplified by Multiclamp 700B and digitized by Axon Digidata 1440a. The data were offline analyzed by Axon pClamp 10 (Molecular Devices). The amplitude of the evoked field response was measured as previously described (Huang et al., 2008).

### *In vitro* experiments

Male and female mice age seven to nine weeks were anesthetized with pentobarbital sodium and decapitated and hippocampal slices prepared for field potential and whole-cell recordings (Pan et al., 2011). The brain was quickly removed and placed in ice-cold buffer containing 110 mm sucrose, 60 mm NaCl, 3 mm KCl, 1.25 mm NaH_2_PO_4_, 28 mm NaHCO_3_, 0.5 mm CaCl_2_, 7.0 mm MgCl_2_, and 5 mm dextrose, saturated with 95% O_2_-5% CO_2_, pH 7.4. Following dissection of hippocampi, transverse slices (400 μm in thickness) were cut with a vibratome and incubated in oxygenated artificial CSF (ACSF) containing 124 mm NaCl, 1.75 mm KCl, 1.25 mm KH_2_PO_4_, 26 mm NaHCO_3_, 2.4 mm CaCl_2_, 1.3 mm MgCl_2_, and 11 mm dextrose for at least 1 h at 32–34°C before recording. The slices were then transferred to a recording chamber mounted on a Zeiss Axioskop2 FS Plus upright microscope.

A bipolar tungsten stimulating electrode was placed near the junction of the granule cell layer and hilus near the midpoint of the suprapyramidal blade of the dentate. Synaptic responses were filtered at 2 kHz and digitized at 5 kHz. Extracellular recordings were obtained with a glass micropipette filled with 2 m NaCl, 2–6 Mil resistance, placed in stratum lucidum near the junction of CA3a and CA3b. Following placement of the extracellular recording electrode, whole-cell recordings of CA3 pyramidal cells visualized by infrared differential interference microscopy were performed in some experiments. Whole-cell recordings of CA3 pyramidal cells in CA3b were established using a glass micropipette filled with the following solution: 140 mm K-gluconate, 10 mm HEPES, 1 mm EGTA, 4 mm NaCl, 4 mm MgATP, 0.3 mm MgGTP, and 14 mm phosphocreatine (pH 7.25). D,L-2-amino-5-phosphonovaleric acid (D,L-APV; 100 μm) was included in the perfusion solution to eliminate contamination of associational-commissural afferents (Maccaferri et al., 1998). Series resistances ranged from 7 to 15 MΩ and were monitored throughout the experiment and not compensated. Experiments were discontinued if the series resistance increased by >20%. Data were collected from slices at room temperature using a Multiclamp 700A amplifier and pClamp 10 software (Molecular Devices). Synaptic events in slices from wild-type mice were evoked by a stimulus pulse (0.2-ms square-wave pulses delivered at 0.03 Hz with a DS3 Digitimer constant-current stimulator). Synaptic events in slices from *DGC ChR* mice were evoked by optogenetic stimulation of granule cells with a 200-μm diameter optical fiber (Thorlab). EPSCs were collected at −65 mV) and IPSCs were collected at 0 mV. The area of at least five stimulus-evoked (0.033 Hz) EPSCs and IPSCs for a given cell was determined and the excitation/inhibition (E/I) ratio for that cell was calculated by dividing the average of EPSCs by the average of IPSCs.

In order for the CA3 pyramidal cell EPSC or fEPSP to be considered evoked by mossy fibers, the following criteria were met: (1) the ratio for paired-pulse facilitation (PPF) at a 60-ms interval was 1.75 or greater; (2) frequency facilitation at 20 Hz was 2.0 or greater as determined by the ratio of the amplitude of the response to the third pulse compared with that to the first pulse (Toth et al., 2000); and (3) application of the DCG-IV (1 μm) at the end of the experiment reduced the amplitude of the evoked EPSC or fEPSP by at least 70%.

LTP of the mossy fiber synapse with CA3 pyramidal cells was evoked by high-frequency stimulation (HFS) consisting of four trains (each train 100 Hz for 1 s) applied at 10-s intervals. LTP was defined as the mean percent increase of EPSC or fEPSP amplitude 10–20 min after HFS relative to that 10 min immediately preceding the HFS. Paired-pulse ratio (PPR) was calculated as the amplitude of the second synaptic response divided by the amplitude of the first synaptic response. The numbers reported for whole-cell or fEPSP recordings in the text, and figure legends reflect the number of animals; in slices from approximately half of animals, recordings were obtained from more than one cell, in which case the results were averaged and reported as a single value for the animal.

Limited availability of *DGC ChR* mice necessitated use of electrical stimulation to activate the mossy fibers for most *in vitro* studies as outlined in the preceding paragraphs. That said, a subset of experiments presented in Figures 3, 4 used optical stimuli (*n* = 3 PBS and *n* = 3 KA for each figure); results for experiments using optical and electrical stimulation were similar, and results have been pooled for presentation.

### Statistical analyses

**Figure 1.**
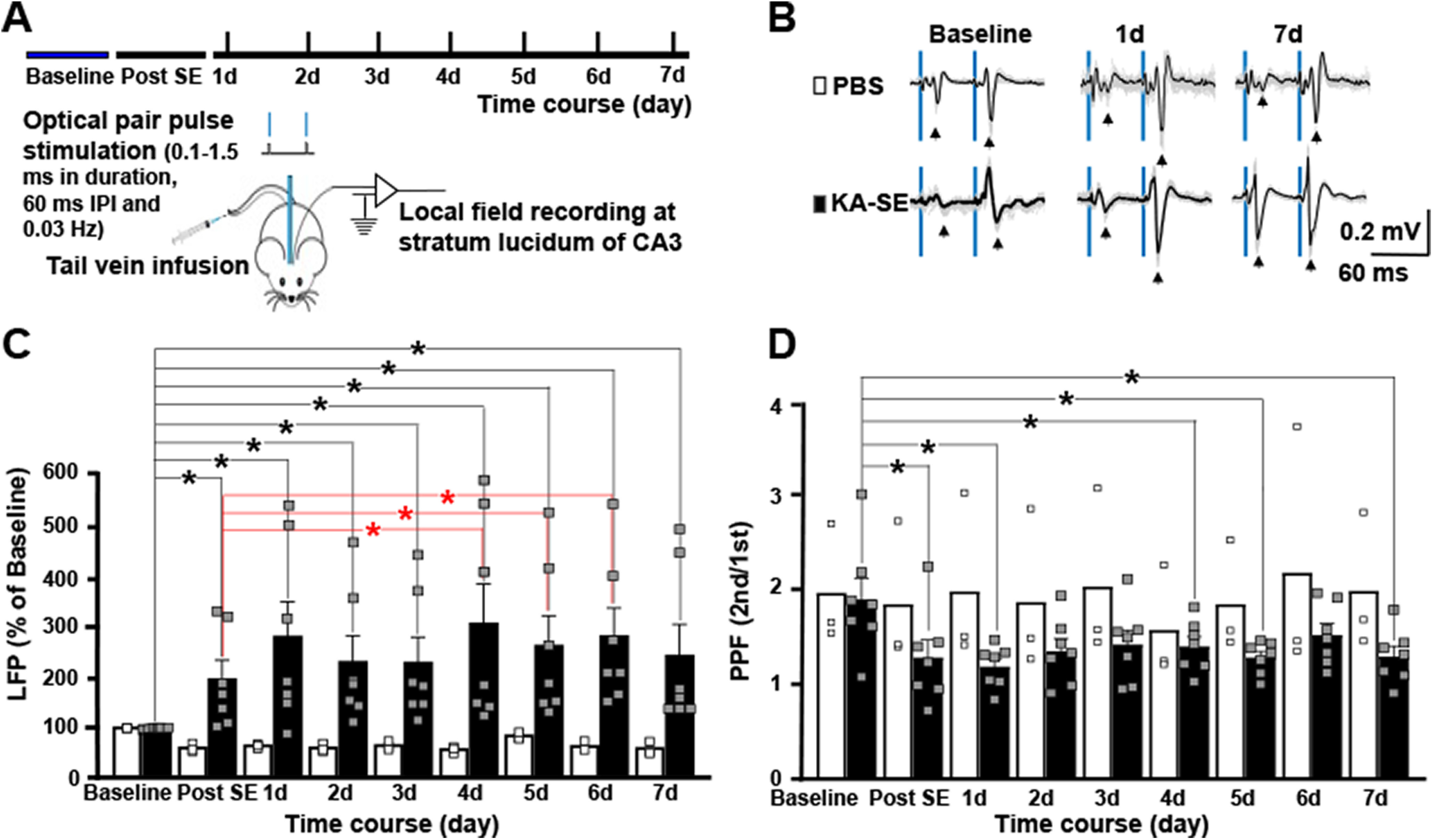
Status epilepticus induces long-lasting potentiation of mossy fiber evoked field potentials in hippocampal region CA3 *in vivo*. ***A***, Schematic presents design of experiment in which optical stimulation of dentate granule cell mossy fibers evokes field potentials recorded in the ipsilateral CA3 region in awake, freely moving adult mice. Following baseline recordings collected for 2 d, animals underwent infusion of KA (16 mg/kg; *n* = 7) or PBS (*n* = 3). Field potentials evoked by pairs of optical stimuli (60-ms interval between stimuli) were recorded several hours later (“post-SE”) and at daily intervals for the following 7 d. ***B***, Representative field potential recordings are presented for PBS and KA-treated animals at baseline, 1 and 7 d following infusion. Blue bars denote the light stimulations. Individual traces are gray and average of traces are black. ***C***, Each of seven animals infused with KA exhibited striking increases in amplitude of evoked field potential detected several hours after status epilepticus which persisted during the following 7 d. By contrast, small reductions in the amplitude of evoked field potentials were detected in each of three animals undergoing infusion of PBS. Bars reflect the mean ± SEM; small squares reflect values of individual animals. Two-way repeated measures ANOVA with *post hoc* paired *t* tests revealed significant differences compared with baseline designated by black asterisks for both PBS and KA groups; red asterisks denote significant increases of amplitude of evoked field potentials in the KA group at days 4–6 compared with measures several hours after SE (post-SE). ***D***, Similar statistical analyses were performed for the PPR data and significant reductions were detected at the post-SE time point in the KA group (paired *t* test compared with baseline *p* = 0.0007) as denoted by black asterisk. Although significant reductions compared with baseline in the KA group were also detected at days 1, 4, and 5 following SE, the magnitude of reduced PPRs was maximal immediately post-SE and further reductions were not observed. Apart from a reduction on day 4 (paired *t* test *p* < 0.003), no changes of PPR were detected in the PBS group.

**Figure 2.**
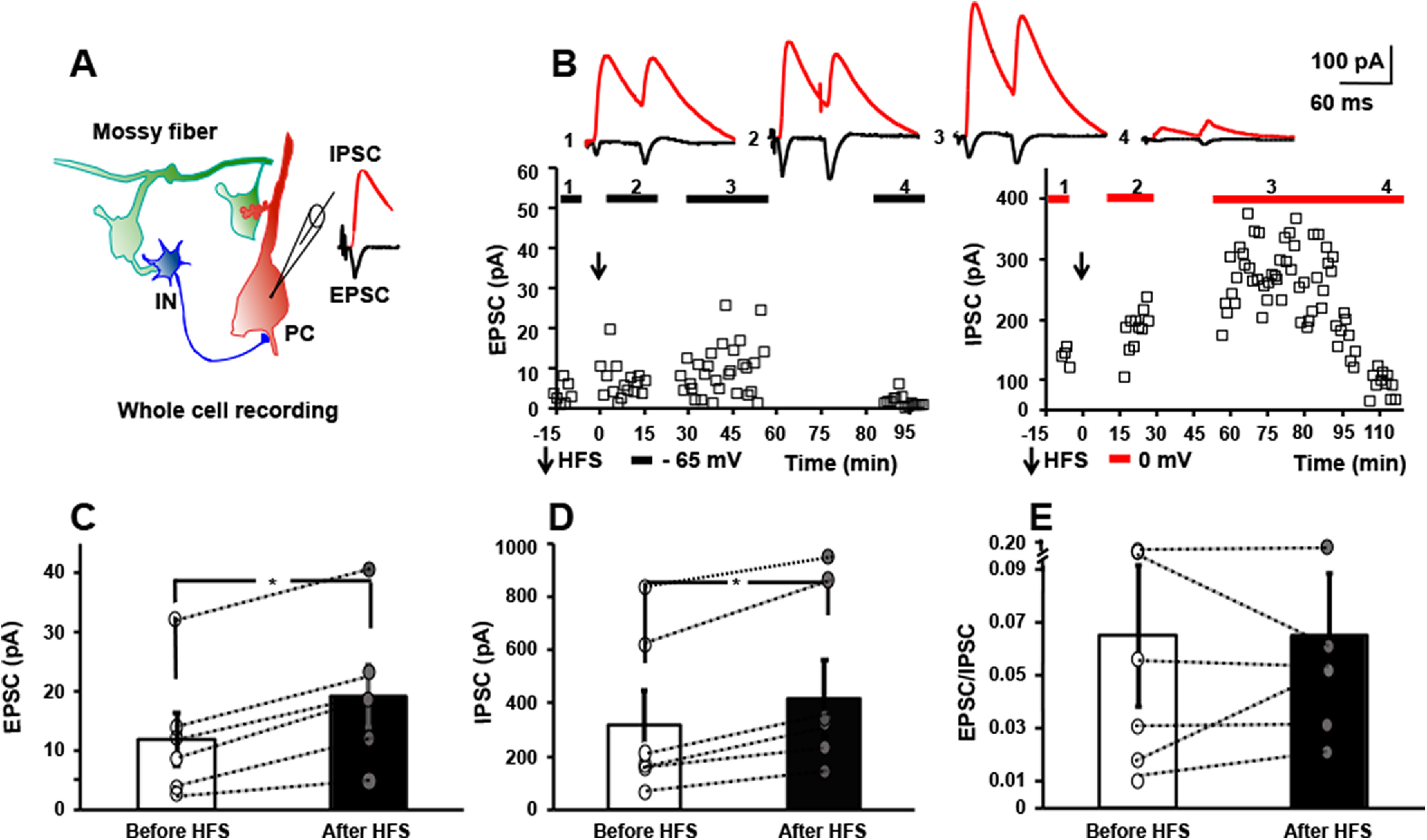
HFS of mossy fibers *in vitro* induces LTP of both monosynaptic EPSC and disynaptic IPSC. ***A***, Schematic of local circuit (left) in which activation of granule cell evokes monosynaptic EPSC (black) and disynaptic IPSC (red) recorded in CA3 pyramidal cell (right). ***B***, Responses of a CA3 pyramidal cell evoked by mossy fiber stimuli (0.033 Hz) in which EPSC (left) and IPSC (right) were collected during baseline recordings at holding potentials of −65 and 0 mv, respectively. Mossy fibers underwent HFS (denoted by arrow) at holding potential of 0 mv and EPSCs collected at holding potential of – 65 mv between 0 and 15 min and again at 30–45 min later; IPSCs were collected at holding potential of 0 mv between 15 and 30 and again between 60 and 90 min after HFS. Between 90 and 115 min, EPSCs were recorded at −65 mv and IPSCs at 0 mv in the presence of DCG-IV (1 μm). Top, Representative traces show individual EPSC and IPSC collected during baseline (1), between 0 and 30 min after HFS (2), between 30 and 90 min after HFS (3), and between 90 and 115 min after HFS in the presence of DCG-IV (1 μm). ***C***, Results of individual cells collected before and after HFS reveal LTP of mossy fiber-CA3 EPSC (58% increase, paired *t* test, *p* = 0.006). ***D***, Results of individual cells collected before and after HFS reveal LTP of mossy fiber-CA3 IPSC (31% increase, paired *t* test, *p* = 0.005). ***E***, E/I ratio of the six cells before versus after HFS reveals values of 0.06 ± 0.03 before HFS, 0.06 ± 0.02 after HFS, paired *t* test, *p* = 0.5.

**Figure 3.**
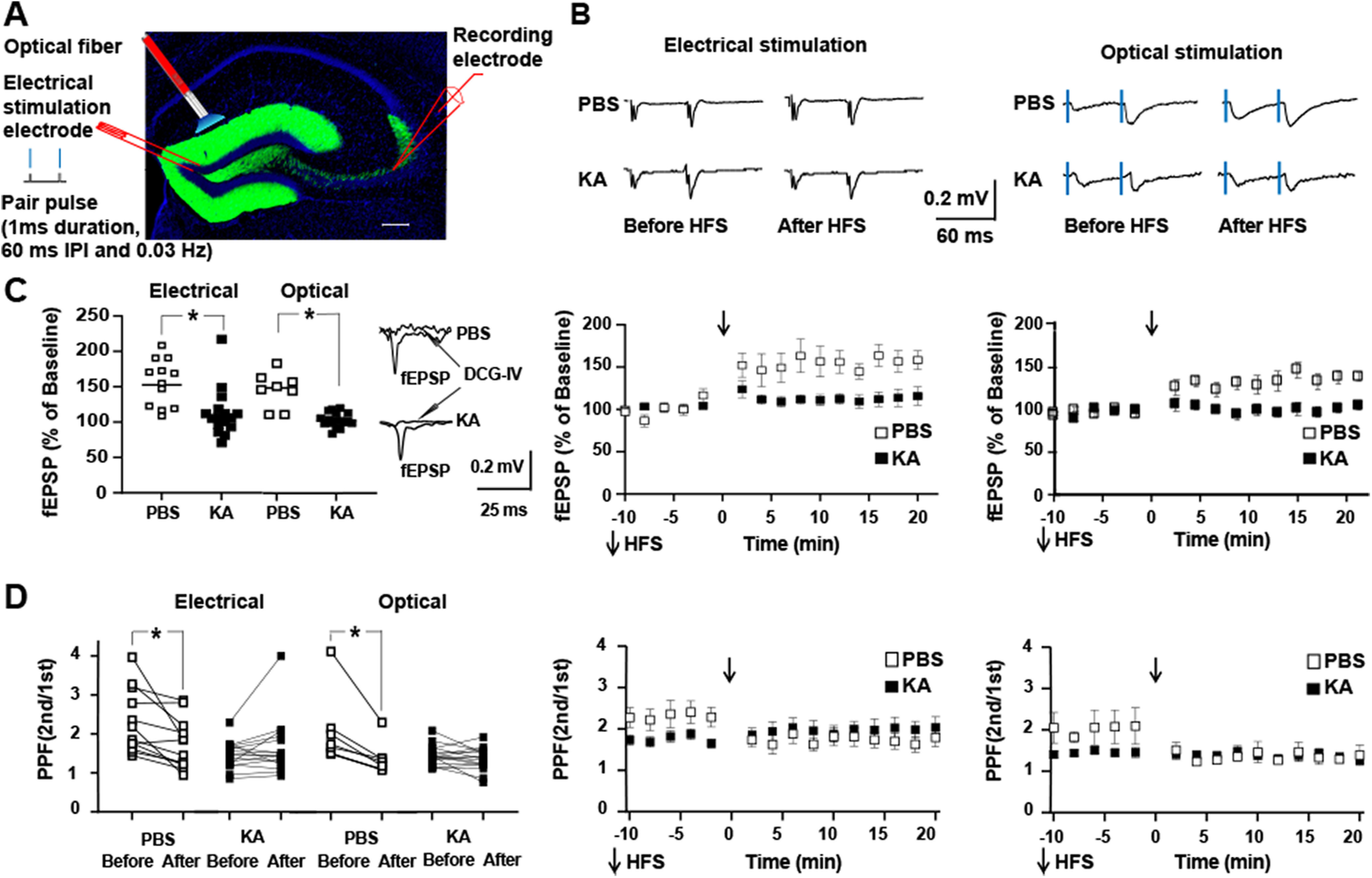
Status epilepticus induces reduction of PPF and reduction of *in vitro* LTP of mossy fiber CA3 fEPSP. ***A***, GFP fluorescence (green) in coronal section of dorsal hippocampus of *DGC ChR* mouse costained with DAPI reveals signal restricted to apical dendrites, cell bodies, and mossy fiber axons of dentate granule cells. Schematic depicts location of optical fiber and stimulating electrode. Stimuli administered in pairs (IPI denotes interpulse interval of 60 ms) at frequency of 0.03 Hz. ***B***, Representative traces show EPSPs evoked by electrical (left) or optical (right) stimulation at time points collected 10 min before and between 10 and 20 min after application of HFS in slices isolated from mice infused with either PBS or KA. Artifact of electrical stimulus was subtracted from tracings. ***C***, left panel, Repeated measures ANOVA with *post hoc* Bonferroni’s revealed that electrical stimulation induced LTP of mossy fiber evoked fEPSP in slices from PBS controls (156 ± 10%, *n* = 12, *post hoc p* = 0.001) but not in slices from KA-treated animals (113 ± 8%, *n* = 16, *post hoc p* = 0.4). Repeated measures ANOVA with *post hoc* Bonferroni’s revealed that optical stimulation induced LTP of mossy fiber evoked fEPSP in slices from PBS controls (141 ± 8%, *n* = 8, *post hoc p* =0.0002) but not in slices from KA-treated animals (100 ± 2.4%, *n* = 17, *post hoc p* = 0.5). Panels in middle and right present time course and mean ± SE of electrical and optimal stimulation, respectively. ***D***, left panel, Repeated measures ANOVA with *post hoc* Bonferroni’s multiple comparisons revealed that electrical stimulation induced reduction of PPF in slices from PBS controls (before HFS 2.3 ± 0.25; after HFS 1.90 ± 0.27, *p* = 0.004) but not from KA-treated animals (before HFS 1.73 ± 0.10; after HFS 2.02 ± 0.22, *p* = 0.03). Likewise repeated measures ANOVA with *post hoc* Bonferroni’s multiple comparisons revealed that optical stimulation induced reduction of PPF in slices from PBS controls (before HFS 1.94 ± 0.23; after HFS 1.42 ± 0.14, *p* = 0.01) but not from KA-treated animals (before HFS 1.44 ± 0.06; after HFS 1.34 ± 0.07, *p* = 0.07). Central and right panels present time course and mean ± SE of electrical and optical stimulation, respectively. *Post hoc* Bonferroni’s multiple comparisons test revealed significant differences of PPF before HFS between PBS and KA undergoing either electrical (*p* = 0.02) or optical stimulation (*p* = 0.01).

**Figure 4.**
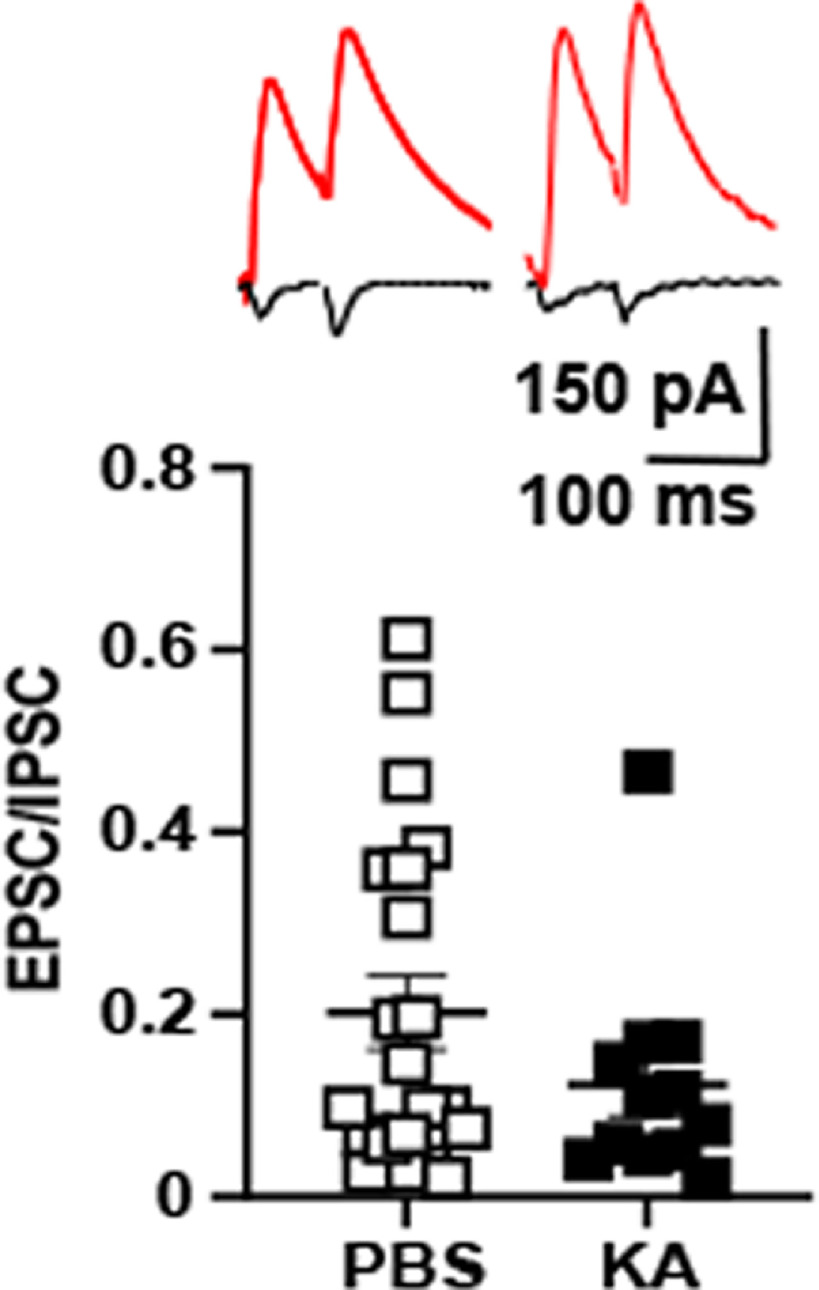
EPSC/IPSC (E/I) ratio reveals nonsignificant reduction in KA compared with PBS infused animals. Top panels show responses of a CA3 pyramidal cell evoked by mossy fiber stimuli (0.033 Hz) in which EPSC (black) and IPSC (red) were collected during baseline recordings at holding potentials of −65 and 0 mv, respectively, from PBS (left) and KA (right) infused animals. Bottom panels present E/I ratio for each cell; the ratio for each of 12 (KA) and 21 (PBS) cells was averaged to obtain the values presented in this figure (KA 0.12 ± 0.03, *n* = 12) compared with PBS infused animals (0.20 ± 0.04, *n* = 21), *p* = 0.14, Student’s *t* test. Synaptic responses induced by optical stimulation of mossy fibers were obtained in three PBS and three KA-treated animals; electrical stimulation was used in the remainder.

**Figure 5.**
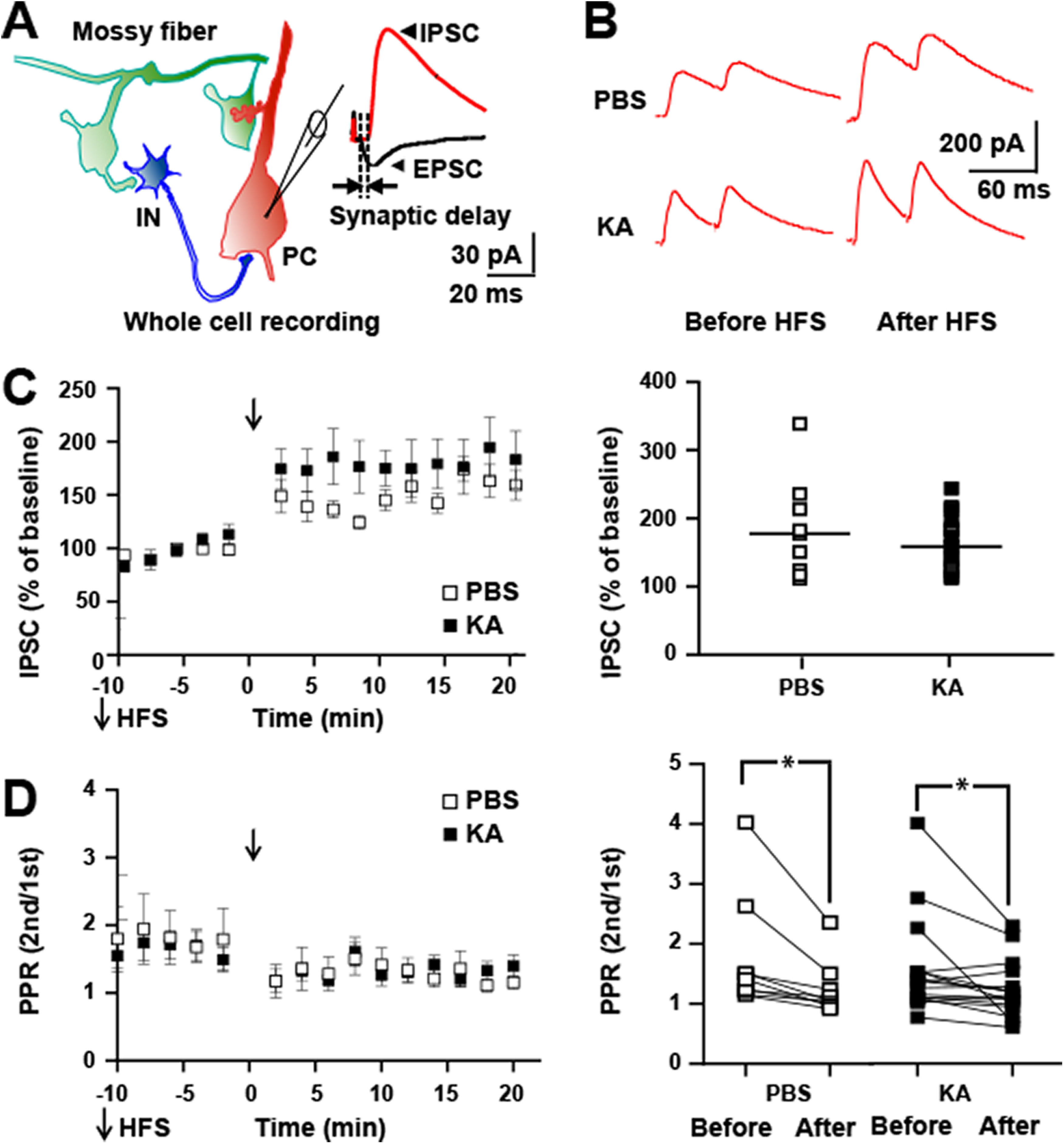
HFS of mossy fibers *in vitro* induces LTP of feedforward IPSC in both controls and following status epilepticus. ***A***, Schematic of local circuit (left) in which activation of granule cell evokes monosynaptic EPSC and disynaptic IPSC recorded in CA3 pyramidal cell (right). Note the delay between onset of EPSC and IPSC approximates 2.5 ms similar to Torborg et al. (2010). ***B***, top, Representative traces show individual IPSCs recorded at holding potential of 0 mv collected 10 min before and between 10 and 20 min after application of HFS in slices isolated from PBS or KA infused mice. ***C***, left, HFS (denoted by arrow) produced LTP of mossy fiber-CA3 disynaptic IPSC in slices from both PBS (184 ± 24%, *n* = 9, *p* = 0.0001, *post hoc* Bonferroni’s) and KA (164 ± 9%, *n* = 17, *p* = 0.0001, *post hoc* Bonferroni’s) infused animals. Right, Results of individual cells are plotted. ***D***, PPR of mossy fiber evoked IPSC of experiment of ***C*** above. Repeated measures ANOVA revealed a *p* value of 0.0001. *Post hoc* Bonferroni’s revealed no significant differences between PBS and KA either before or after HFS. *Post hoc* Bonferroni’s revealed a significant reduction of PPR following HFS in both the PBS (1.76 ± 0.3 before, 1.24 ± 0.1 after, *n* = 9, *p* = 0.02) and KA (1.59 ± 0.2 before, 1.26 ± 0.1 after, *n* = 17, *p* = 0.04) groups.

Data are presented as means ± SE as well as individual animals (Fig. 1), individual cells (Figs. 2, 4, 5), and individual slices (Fig. 3); only a single cell (Figs. 2, 4, 5) or single slice (Fig. 3) was obtained from a given animal in the vast majority of experiments. PPRs were calculated as the mean of the second response divided by the mean of the first response (Kim and Alger, 2001). Data were collected from both male and female animals. Sample sizes were estimated from results of prior studies (Pan et al., 2019). Data were analyzed by repeated measures two-way ANOVA and a *post hoc* Bonferroni test with the exception of data in Figure 2 in which the *post hoc* test was paired *t* test.

## Results

### Status epilepticus induces long-lasting potentiation of mossy fiber evoked field potentials in hippocampal region CA3 *in vivo*

Knowledge that the granule cells fire in bursts exceeding 100 Hz for tens of seconds during limbic seizures (Labiner et al., 1993) led us to ask whether an episode of status epilepticus modified mossy fiber evoked field potentials recorded in hippocampal region CA3 *in vivo*. To address this question, mossy fibers were activated optogenetically, an approach made possible by crossing Dock 10 Cre with floxed channelrhodopsin-2 mice (*DGC ChR*) which express channelrhodopsin-2 within hippocampus exclusively in the dentate granule cells and their mossy fiber axons (Fig. 3*A*). An optical fiber was placed in the suprapyramidal granule cell layer to enable optogenetic activation of the granule cells *in vivo* (Figs. 1*A*, 3*A*). Local field potential responses evoked by light stimuli were assessed in recording electrodes in hippocampal region CA3 ipsilateral to the optical fiber (Fig. 1*A*,*B*). Pairs of stimuli (60-ms interval) delivered at low frequency (0.033 Hz) evoked field potentials, the amplitude of the second response markedly greater than the first (Fig. 1*B*); the robust PPF is characteristic of mossy fiber evoked synaptic responses previously demonstrated *in vivo* using bulk electrical or optogenetic stimulation (Hagena and Manahan-Vaughan, 2010; Zucca et al., 2017).

The effects of status epilepticus on mossy fiber evoked field potentials were assessed in the following experiments. Stability of light evoked local field potentials assessed in awake, freely moving animals was established in input output curves conducted for at least 2 d before induction of status epilepticus. Infusion of the chemoconvulsant, KA (16 mg/kg), into the tail vein evoked status epilepticus evidenced by repetitive behavioral seizures beginning during and immediately following infusion and persisting for ∼1 h before remitting spontaneously. The seizure behaviors ranged from episodes of immobilization and head nodding (Racine classes 1–2) to rearing and falling with clonic movements of forelimbs (Racine classes 4–5; Drysdale et al., 2021). Increases in the amplitude of the optically evoked response were evident several hours following status epilepticus in each of the seven animals studied (197 ± 36%, mean ± SEM of baseline; Fig. 1*C*). In contrast, following infusion of vehicle (PBS) into tail vein, reductions of the amplitude of the evoked potential ranging from 17% to 50% were detected in each of three control animals assessed (Fig. 1*C*). Two**-**way repeated measures ANOVA revealed significant differences between PBS and KA groups (*F*_(5,40)_ = 8.328, *p* = 0.001, two-way ANOVA). *Post hoc* paired *t* tests revealed significant differences of both the PBS and KA groups (*p* = 0.002 and *p* = 0.002, respectively), the directions of changes being decreased in the PBS and increased in the KA cohorts. Interestingly, the amplitude of evoked field potential continued to increase in the days following SE as evident in part by significant increases between measures post-SE compared with days 4–6 (post-SE 197 ± 36% vs 6 d 283 ± 55%, paired *t* test *p* = 0.016).

The increased evoked field potential evident shortly following status epilepticus was accompanied by a significant reduction of PPF in comparison to PBS controls (two**-**way repeated measures ANOVA revealed significant difference between PBS and KA groups (*F*_(8,64)_ = 8.2245, *p* = 0.0001). The PPR in the SE group decreased from baseline value of 1.83 ± 0.22 to 1.12 ± 0.18 at the post-SE time point (*post hoc* paired *t* test *p* = 0.007; Fig. 1*D*). In contrast to the increased amplitude of the optically evoked field during the days following SE, no further reductions of PPF were observed following the post-SE time point (Fig. 1*D*). No significant changes of PPR were detected in the PBS controls save for a reduction on day 4 (Fig. 1*D*, paired *t* test *p* < 0.003).

In sum, status epilepticus induced a striking increase of the mossy fiber evoked field potential in CA3. Mossy fiber evoked field potentials detected in CA3 *in vivo* likely include a non-NMDA receptor EPSP (Nicoll and Schmitz, 2005) and potentially an IPSP (Glickfeld et al., 2009; Bazelot et al., 2010) and NMDA receptor EPSP (Kwon and Castillo, 2008). This led us to examine the effects of HFS of mossy fibers on monosynaptic excitatory and feedforward inhibitory synaptic inputs to CA3 pyramidal cells.

### HFS of mossy fibers induces LTP of both monosynaptic EPSC and disynaptic IPSC

Granule cells in awake, freely moving rodents typically fire at low rates (<0.5 Hz) but exhibit bursts of 5–10 action potentials at frequencies of 10–40 Hz when the animal enters a place field (Jung and McNaughton, 1993; Henze et al., 2002; Vandael et al., 2020). By contrast, granule cells fire repeated bursts of action potentials at frequencies exceeding 100 Hz over periods lasting tens of seconds during hippocampal seizures (Labiner et al., 1993). We asked whether stimulating mossy fibers with a pattern mimicking activity during a seizure modified the efficacy of its synaptic inputs to CA3. Shortly (∼2.5 ms) following onset of the monosynaptic EPSC, mossy fibers evoke a feedforward IPSC (Fig. 2*A*); its short latency together with its persistence following tetanus toxin mediated inhibition of glutamate release from CA3 pyramidal cell axons (Torborg et al., 2010) demonstrates that this IPSC is mediated by feedforward rather than recurrent connections.To examine the effects of sustained high-frequency firing of mossy fibers on synaptic responses. hippocampal slices were isolated and whole-cell recordings of CA3 pyramidal cells established. Mossy fiber EPSCs were evoked at a holding potential of –65 mV (0.033 Hz) after which the holding potential was shifted to 0 mv and IPSCs evoked. Following collection of baseline data, mossy fibers were stimulated electrically with a 1-s train of 100-Hz pulses which was repeated three additional times at 10-s intervals. The trains were administered at a holding potential of either −65 (*n* = 3) or 0 (*n* = 3) mv (Fig. 2*B*,*C*); similar results were obtained when trains were administered at either holding potential (data not shown). Following the four trains, EPSCs were measured for the next 15 min at a holding potential of – 65 mv at which time holding potential was shifted to 0 mv and IPSCs were measured for the next 15 min. HFS of the mossy fibers induced significant potentiation of the EPSC (before HFS 12 ± 4 pA, after HFS 19 ± 5 pA, *n* = 6, *p* = 0.006, paired *t* test; Fig. 2*B*,*C*), confirming previous findings of multiple investigators (Nicoll and Schmitz, 2005). Like the EPSC. HFS of the mossy fibers induced potentiation of the mossy fiber evoked disynaptic IPSC (before HFS 319 ± 130 pA, after HFS 418 ± 144 pA, 31%, *n* = 6, *p* = 0.005, paired *t* test; Fig. 2*B*,*D*). Before HFS, the E/I ratio was 0.06 ± 0.03, reflecting the markedly higher amplitude of the IPSC. Following HFS, the E/I ratio remained 0.06 ± 0.02 (paired *t* test, *p* = 0.15) reflecting the HFS-induced enhancement of both EPSC and IPSC.

### Effects of status epilepticus on mossy fiber evoked monosynaptic excitatory responses of CA3 pyramidal cells: field potential analyses

The long-lasting potentiation of mossy fiber evoked field potentials in CA3 detected *in vivo* led us to hypothesize that the non-NMDA mediated monosynaptic excitatory synapse had undergone LTP *in vivo.* This synapse exhibits a low probability of release evidenced in part by robust facilitation of the response evoked by the second of a pair of stimuli applied at a short interval (Nicoll and Schmitz, 2005). LTP of this synapse is because of enhanced probability of release of glutamate, evidenced in part by reduction of PPR. If status epilepticus had induced LTP of this synapse *in vivo,* analysis of this synapse in slices isolated from these animals would be expected to reveal: (1) a reduction of the PPR; and (2) reduced magnitude of LTP induced by mossy fiber stimulation. To test these predictions, hippocampal slices were isolated 5–7 d following KA-induced status epilepticus and compared with controls undergoing infusion of PBS. Responses to activation of the mossy fibers by electrical or optogenetic stimulation were examined with a field potential recording electrode in stratum lucidum (Fig. 3*A*,*B*). APV was included in the media to enable study of the non-NMDA receptor synapse.

A reduction of PPR was evident in slices isolated following status epilepticus in comparison to PBS controls. Mossy fibers were stimulated electrically in some experiments and optically in others (Fig. 3). Marked PPF was evident in slices from PBS controls in which two stimuli were applied at a short (60 ms) interval (electrical 2.3 ± 0.25, *n* = 12, mean ± SE; optical 1.94 ± 0.25, *n* = 8). By comparison, a reduction of the magnitude of PPF was evident in slices isolated following status epilepticus when mossy fibers were stimulated electrically (1.73 ± 0.10, *n* = 16, repeated measures two-way ANOVA with *post hoc* Bonferroni’s PBS vs KA *p* = 0.02) or optically (1.44 ± 0.06, *n* = 17, repeated measures two-way ANOVA with *post hoc* Bonferroni’s PBS vs KA *p* = 0.01).

Following assessment of PPR, plasticity induced by HFS of mossy fibers was examined. In slices isolated from controls, HFS of the mossy fibers induced marked potentiation of the mossy fiber-CA3 fEPSP (150 ± 6.7%, *n* = 20; Fig. 3*B*,*C*) . By contrast, in slices isolated following status epilepticus, HFS of the mossy fibers induced a minimal potentiation of the mossy fiber-CA3 fEPSP (106 ± 4.4%, *n* = 33; Fig. 3*B*,*C*). Repeated measures two-way ANOVA with *post hoc* Bonferroni’s revealed significant differences in the magnitude of LTP of PBS compared with KA (*p* = 0.0001).

Effects of HFS of the mossy fibers on PPR also diverged in slices from controls and status epilepticus. LTP in slices of PBS controls was paralleled by significant reduction of PPR (before, 2.3 ± 0.2; after 1.7 ± 0.2; *n* = 20; Fig. 3*D*, *p* = 0.0001, paired *t* test). In sharp contrast, no significant change of PPR was detected following HFS in slices isolated following status epilepticus (before 1.60 ± 0.1; after 1.71 ± 0.1, *n* = 33; Fig. 3*D*, *p* = 0.999, paired *t* test).

In sum, the effects of status epilepticus on this synapse were threefold: (1) before HFS *in vitro*, there was a significant reduction of PPF following status epilepticus in comparison to PBS controls; (2) the magnitude of the HFS induced potentiation of the fEPSP was reduced following status epilepticus in comparison to controls; (3) HFS induced a reduction of PPF in slices from PBS treated animals but not following SE. Collectively, these findings are consistent with the idea that status epilepticus induced LTP of this excitatory synapse *in vivo* and this partially occluded potentiation by HFS *in vitro*.

### Effects of status epilepticus on mossy fiber-evoked E/I ratio

The evidence that status epilepticus induced LTP of the mossy fiber CA3 synapse led us to ask whether it also induced LTP of feedforward inhibition. If so, then the ratio of the mossy fiber evoked EPSC and IPSC should be similar in hippocampal slices isolated following status epilepticus in comparison to PBS controls. To address this question, hippocampal slices were isolated from animals following infusion of PBS or KA and whole-cell recordings of CA3 pyramidal cells established. Mossy fiber EPSCs were evoked by electrical or optogenetic stimulation at a holding potential of –65 mV after which holding potential was shifted to 0 mv and IPSCs evoked. The magnitude of the evoked IPSC markedly exceeded the EPSC in slices from PBS controls (E/I ratio of 0.20 ± 0.04, *n* = 21; Fig. 4). The predominance of inhibition was maintained in slices isolated following status epilepticus evidenced by an E/I ratio of 0.12 ± 0.03 (*n* = 12; Fig. 4); the reduction compared with PBS was not significant (*p* = 0.09, unpaired *t* test). Together with evidence of LTP of the monosynaptic EPSC, the nonsignificant trend to a lower E/I ratio following status epilepticus indicates that that the mossy fiber-CA3 disynaptic IPSC had also undergone LTP *in vivo.*

### Effects of status epilepticus on mossy fiber-evoked disynaptic inhibitory responses of CA3 pyramidal cells

One consequence of status epilepticus-induced LTP of the mossy fiber-CA3 EPSC was the reduction of LTP in response to subsequent HFS of the mossy fibers in slices *ex vivo.* Here, we asked whether LTP of disynaptic inhibition was similarly reduced following status epilepticus. To address this question, hippocampal slices were isolated from animals following infusion of PBS or KA and whole-cell recordings of CA3 pyramidal cells established. Mossy fiber IPSCs were evoked at a holding potential of 0 mV. HFS of the mossy fibers induced marked potentiation of the mossy fiber evoked disynaptic IPSC in slices isolated from PBS control animals (184 ± 24%, *n* = 9, *post hoc* Bonferroni’s *p* = 0.0001; Fig. 5*B*,*C*). Unlike the mossy fiber evoked EPSC, HFS of the mossy fibers also induced marked potentiation of the IPSC in slices isolated following status epilepticus (164 ± 9%, *n* = 17, *p* = 0.0001; Fig. 5*B*,*C*). Thus, HFS induced significant increases of the IPSC in slices from both groups; no significant differences between the groups were detected either before or after HFS (repeated measures ANOVA; *F*_(24,24)_ = 1.221, *p* = 0.3, repeated measures two-way ANOVA). HFS induced reductions of PPR in slices isolated from both the PBS and KA groups (Fig. 5*B*,*D*); no significant differences were detected between PBS and KA groups either before or after HFS (Fig. 5*B*,*D*).

### Effects of status epilepticus on intrinsic properties of CA3 pyramidal cells

The plasticities of both excitatory and inhibitory synaptic inputs led us to ask whether status epilepticus also induced modifications of intrinsic properties of CA3 pyramidal cells. No significant differences in any of the intrinsic properties were detected (Table 1).

**Table 1:** Intrinsic properties of CA3 pyramidal cells following status epilepticus

	PBS	KA	*p* values
Rm (MΩ)	105.4 ± 6.7	115.8 ± 5.2	0.133
RMP (mV)	63.1 ± 0.6	61.8 ± 1.0	0.131
MC (pF)	23.9 ± 4.5	18.0 ± 2.1	0.160
TC (MS)	2.23 ± 0.31	2.18 ± 0.24	0.454
Latency to first AP (ms)	161 ± 68.8	133 ± 50	0.367
AP threshold (mV)	39.7 ± 2.9	42.3 ± 1.0	0.27
AP amplitude (mV)	58.7 ± 6.2	68.4 ± 6.2	0.144
AP half width (ms)	2.99 ± 0.26	2.58 ± 0.3	0.142
Afterhyperpolarization potential (mv)	3.6 ± 1.4	5.9 ± 1.5	0.146

Intrinsic properties of CA3 pyramidal cells following status epilepticus (KA, *n* = 11) or controls (PBS, *n* = 15). Membrane resistance (MΩ), resting membrane potential (RMP), membrane capacitance (MC), time constant (TC), action potential (AP) threshold, amplitude, and half width are presented as mean ± SE. No significant differences were observed as evident from *p* values of Student’s *t* test.

## Discussion

We investigated the hippocampal mossy fiber CA3 pyramidal cell microcircuit in models of TLE spanning *in vitro*, *in vivo*, and *ex vivo* preparations. Expression of channelrhodopsin-2 in the dentate granule cells of *DGC ChR* mice enabled the selective activation of mossy fiber axons *in vivo* in a model of TLE caused by status epilepticus. Principal findings included the following. (1) *In vivo* studies revealed marked potentiation of mossy fiber evoked field potentials in hippocampal CA3 beginning within hours following status epilepticus, a potentiation which persisted at least 7 d. (2) Stimulation of mossy fibers in hippocampal slices *in vitro* using patterns of activity mimicking seizures induced LTP not only of the monosynaptic EPSC but also of the disynaptic IPSC of CA3 pyramidal cells. (3) *Ex vivo* study of hippocampal slices isolated from animals undergoing status epilepticus revealed reduction of PPF of the mossy fiber evoked non-NMDA receptor EPSC of CA3 pyramidal cells and impaired development of LTP. (4) The ratio of mossy fiber evoked EPSC/IPSC in CA3 pyramidal cells was similar in slices from controls and following status epilepticus. (5) Unlike the EPSC, HFS induced LTP of the mossy fiber evoked disynaptic IPSC to a similar extent in slices from controls and following status epilepticus. (6) In contrast to these synaptic plasticities, no changes of intrinsic properties of CA3 pyramidal cells were detected following status epilepticus. We conclude that patterns of granule cell activity mimicking seizures are sufficient to induce LTP of both monosynaptic EPSC and disynaptic IPSC of CA3 pyramidal cells, plasticities found in *ex vivo* studies of animals following status epilepticus. We suggest that activation of dentate granule cells during seizures induces these plasticities *in vivo* and the retained balance of synaptic excitation and inhibition limits excessive activation of CA3 pyramidal cells, thereby protecting animals from spontaneous recurrent seizures at this interval following status epilepticus.

Our *in vivo* studies revealed striking potentiation of the mossy fiber evoked local field potential in CA3 accompanied by a reduction of PPF evident within a few hours of status epilepticus. These findings are likely because of LTP of the mossy fiber CA3 non-NMDA receptor EPSP, the reduction of PPF reflecting the enhanced probability of glutamate release underlying LTP of this synapse (Nicoll and Schmitz, 2005). The potentiation of the mossy fiber evoked LFP continued to evolve over the next several days as evidenced by significant increases of the evoked LFP on days 4–6 when compared with measures several hours after SE (post-SE group; Fig. 1). Although the magnitude of the evoked LFP continued to increase during this interval, no further reduction of PPR was observed. This could reflect the emergence of a postsynaptic NMDA receptor dependent LTP (Kwon and Castillo, 2008). The fact that IPSPs can generate field potentials in hippocampal recordings (Glickfeld et al., 2009; Bazelot et al., 2010) raises the possibility that potentiation of mossy fiber evoked IPSPs contributed to these field potentials.

*Ex vivo* studies of these synapses in slices isolated from animals following status epilepticus support the idea that the mossy fiber CA3 non-NMDA receptor EPSP did indeed undergo LTP *in vivo*, as evidenced by reduced PPF as well as inability to induce LTP with HFS of mossy fibers *in vitro.* The magnitude of reductions of LTP and PPF are similar to earlier studies of the mossy fiber/CA3 synapse following prolonged seizures in rats (Goussakov et al., 2000), the present findings confirming and extending this work. The similarity in the ratio of the monosynaptic EPSC and disynaptic IPSC in slices from controls and following status epilepticus implies that the disynaptic IPSC also underwent LTP *in vivo.* Previous *in vitro* studies revealed activity dependent plasticities of both the monosynaptic EPSC and as well as disynaptic IPSC of CA3 pyramidal cells evoked by mossy fiber stimulation. Brief bursts of granule cells (20 action potentials at 150 Hz) induce a brief and preferential enhancement of disynaptic IPSC of CA3 pyramidal cells persisting for ∼10 s (Neubrandt et al., 2018). Activating mossy fibers in cultured hippocampal slices with more prolonged physiological patterns induced increases of both the monosynaptic EPSC and disynaptic IPSC of CA3 pyramidal cells for ∼10 min (Mori et al., 2007), results similar to the long-lasting plasticities observed here. We suggest that the pathologic activity of dentate granule cells during status epilepticus induced these plasticities, a suggestion based on sufficiency of similar activity to induce these plasticities in acutely isolated slices (Fig. 2) together with evidence of sustained high-frequency firing of granule cells during limbic seizures *in vivo* (Labiner et al., 1993).

What are the mechanisms by which status epilepticus induces these synaptic plasticities? With respect to the excitatory synapse, we suggest that events intrinsic to the mossy fiber bouton mediate induction of non-NMDA receptor LTP of this synapse, a suggestion supported by enhanced release of glutamate from mossy fiber synaptosomes correlating with occlusion of LTP following status epilepticus (Goussakov et al., 2000). Chemical genetic methods established a requirement of the BDNF receptor, TrkB, for induction of non-NMDA receptor presynaptic LTP induced by mossy fiber stimulation *in vitro* (Huang et al., 2008). Status epilepticus induces activation of TrkB in mossy fiber boutons as evidenced by increased immunoreactivity of a surrogate of activated TrkB (Helgager et al., 2013); it seems plausible that one consequence of status epilepticus-induced TrkB activation in this locale is LTP of this synapse. With respect to LTP of the IPSC, elucidating mechanisms will require identification of the responsible population of interneurons. Anatomic and electrophysiological evidence suggest that parvalbumin interneurons may contribute, evidence including contacts formed by filopodial extensions of mossy fiber boutons with parvalbumin interneurons (Ruediger et al., 2011; Guo et al., 2018). Additional candidates include Ivy cells and CCK basket cells (Neubrandt et al., 2018). Interestingly, striking increases in the number of filopodia per mossy fiber bouton have been identified 48 h following status epilepticus, structural plasticities that persist for at least one month (Danzer et al., 2010). In contrast to occlusion of LTP of the monosynaptic EPSC, HFS of mossy fibers in slices following status epilepticus induced LTP of the disynaptic IPSC. Perhaps the increased numbers of filopodia and presumptive increased numbers of mossy fiber/interneuron synapses underlies the enhanced feedforward inhibition observed days following status epilepticus; if so, the efficacy of individual synapses at this time may be similar to PBS controls and capable of further enhancement in response to HFS.

The pathologic activity-induced enhancement of the principal excitatory synapse connecting dentate granule cells with CA3 pyramidal cells observed here would be expected to impact the operation of CA3 pyramidal cells within its normal range of activity, necessitating implementation of a homeostatic response. The homeostatic mechanism implemented consisted of enhanced synaptic inhibition; no plasticity of intrinsic properties was detected. Homeostatic mechanisms restoring E/I balance mediated by potentiation of inhibitory synapses have been described following experience driven potentiation of excitatory cell assemblies in diverse preparations (for review, see Barron et al., 2017). Analyses of the cellular and synaptic mechanisms of the *Aplysia* siphon withdrawal response revealed activity dependent enhancement of synaptic inhibition, a process thought to contribute to dynamic gain control of the response (Fischer and Carew, 1993). Synaptic inhibition slowly increased in an activity dependent manner to rebalance a large increase of synaptic excitation mediating a shift of preferred auditory stimuli of mouse cortical neurons, the rebalanced E/I ratio contributing to a retuned receptive field (Froemke et al., 2007). Maintaining E/I balance of CA3 pyramidal cells is particularly important in view of their propensity to fire bursts of action potentials together with their extensive recurrent excitatory synapses, rendering these hippocampal neurons highly susceptible to seizures (Miles and Wong, 1983). The danger inherent in this susceptibility is enhanced by the striking increases in the excitability of the dentate gyrus during epileptogenesis and chronic epilepsy related to multiple factors including axonal sprouting (Tauck and Nadler, 1985), alterations of local inhibitory circuits (Kobayashi and Buckmaster, 2003; Yu et al., 2013; Zhang et al., 2009; Sun et al., 2014; Dengler et al., 2017), and neurogenesis (Parent et al., 1997). The net result is enhancement of the dominant excitatory synaptic input to CA3 pyramidal cells. Feedforward inhibition prevented CA3 pyramidal cells from prolonged depolarization and repetitive firing induced by mossy fiber driven EPSPs in hippocampal slices from normal animals (Torborg et al., 2010). It seems likely that a beneficial consequence of the LTP of feedforward inhibition observed here is to limit excessive activation of CA3 pyramidal cells, thereby protecting animals from spontaneous seizures at this interval following status epilepticus. Restated, LTP of mossy fiber evoked feedforward inhibition of CA3 pyramidal cells serves as an additional safeguard against collapse of the dentate gate in the context of TLE. Importantly, continuous video-EEG analyses of animals in the model studied here reveal no seizures during the first week following status epilepticus, the interval during which the current studies were performed (Drysdale et al., 2021). It will be interesting to determine whether impairments of mossy fiber evoked feedforward inhibition accompany emergence of spontaneous recurrent seizures in this model. A molecular understanding of the cellular and synaptic mechanisms underlying formation and expression of this enhanced feedforward inhibition will hopefully reveal valuable targets for preventive and/or disease modifying therapies of TLE.

## Synthesis

Reviewing Editor: Katalin Toth, University of Ottawa Decisions are customarily a result of the Reviewing Editor and the peer reviewers coming together and discussing their recommendations until a consensus is reached. When revisions are invited, a fact-based synthesis statement explaining their decision and outlining what is needed to prepare a revision will be listed below. The following reviewer(s) agreed to reveal their identity: John Josh Lawrence, Simon Chamberland.

Both reviewers agree that the manuscript contains important data and the results are convincing. They both offer several points of improvement.

The mossy fibre events have different parameters from what we see in the published literature. The authors should comment on potential reasons that could explain this difference and give full details on the optogenetic stimulation paradigm and explain why data obtained with electrical and optogenetic stimulation were pooled. Given that these stimulus paradigms can lead to quite different results, these may be better presented separately.

Rev.#1.

In the current manuscript “Long term potentiation of mossy fiber feedforward inhibition of CA3 pyramidal cells maintains E/I balance in epilepsy model", the authors make use of a transgenic mouse to photoactivate dentate granule cells in vivo and in vitro, demonstrating that there is long term potentiation of glutamatergic transmission onto CA3 pyramidal cells that is also accompanied by potentiation of disynaptic inhibition. The authors suggest that E/I balance is maintained to protect the hippocampus against seizures in the latent period following a seizure and before the onset of spontaneous seizure activity. The data is convincing but the manuscript could be further improved by the inclusion of additional methodological details, improved analyses and display of figures, consistent use of statistics, and a more thoughtful use of color in figures.

Major points:

1) P. 5, Optogenetic stimulation details are limited. It is not clear what duration light pulses were. “Modifying TTL pulse duration from 0.5 to 1.5 sec in 0.1 sec steps...” is this correct? The Kohara paper used 2 ms. Fig 1B and all subsequent traces (i.e. Fig. 3B) should feature a blue vertical bar indicating what duration was used. Were in vivo and in vitro experiments comparable in light pulse duration?

2) P.7, The display and possibly analysis of voltage and current traces is insufficient and potentially flawed. In addition, given the traces in Fig. 3C and the stability of the recordings that the authors claim, it should be possible to utilize failures, especially in DCG-4 conditions, to subtract the stimulus artifact. This would greatly improve the display of traces in Fig. 1B, etc.

3) “Paired-pulse ratio (PPR) was calculated as the amplitude of the second synaptic response divided by the amplitude of the first synaptic response.” - this phrasing suggests the introduction of spurious PPF (Kim and Alger 2001).

4) Additional details regarding justification for the dosage of kainic acid are sparse. Perhaps these details are is in the Drysdale paper under review, but it is difficult to evaluate the quality of the seizure experiments without additional information regarding the effectiveness of behavioral seizures.

5) The manuscript could be improved by avoiding conflating the electrical stimulation and optogenetic stimulation datasets, particularly when optical stimulation may be very different than electrical stimulation regarding the number of axonal action potentials elicited per stimulus. Suggest that these experiments be presented separately.

Minor points:

1) Abstract: Please use appropriate, current optogenetic terminology (i.e. channelrhodopsin-2).

2) Although the Discussion does a more thorough job, additional references should be added to the Introduction (i.e. Mori et al. 2007, Vandael et al. 2020).

3) There are some discrepancies regarding use of post-hoc statistics. P. 9 indicates paired t-test but Fig. 1 legend indicates Fisher. But shouldn’t this be Tukey?

4) P.10, middle: This section on granule cell firing in awake, freely moving rodents is outdated. Please update to include Vandael et al. 2020 paper.

5) P.10, p.11: grammatical - please use comma instead of period.

6) P.13, “...and this limited additional potentiation...”; consider using “partly occluded"

7) P.13, the wording of “nonsignificant reduction” (Fig. 4 legend) and “non-significant trend towards a lower E/I ratio” is awkward. Suggest rephrasing this section to feature “inhibition and E/I ratio was maintained"

8) P.14 - refer to Fig. 5A in the text.

9) While the Discussion is well done, the author is left asking why, eventually, E/I balance is lost further in the disease process. Some speculation is warranted, particularly a discussion about how the new data contained in the manuscript sheds light on potential downstream effects that occur later in the disease process.

Figures

1) In general, if the authors want to commit to color figures in one panel, then color should be applied throughout all panels of the figure to improve readability and presentation. For example, use of red in Fig. 1C could be applied more generally to Fig. 1. The use of grey made it easy to miss the legend key for Fig. 1C/D in Fig. 1B - this would not happen if color was applied to all figures.

2) Suggest that 1A and 3A be combined in a Methods figure. Optical stimulation frequency and duration could be added to Fig. 1A schematic for readability and clarity.

3) Suggest Fig. 1B could be improved by adding individual traces in gray with overlaid average in black, and extended for all conditions in Fig. 1C/D.

4) In Fig. 1C, Y axis should be cut off at 400% (but with statistical bars unchanged). Same with Fig. 1D - Y axis should extend to 4, not 6, with statistical bars unchanged. X axis should be 60 ms.

5) Fig 2 - could use the color scheme in Fig 2A to color EPSC and IPSC differently. Suggest horizontal bars indicating conditions to be added to Fig. 2B. Less major tics in Fig. 2C-E.

6) Fig. 5B - more labeling needed. Fig. 5C, Y title - should be IPSC, correct?

Rev. #2.

The authors report that seizures induce long-term synaptic plasticity at the first synapses of the hippocampal tri-synaptic loop, the dentate gyrus mossy fibers (MF) to CA3 projections. By using a combination of optogenetic and electrophysiological recordings in vivo and in vitro, the authors demonstrate that seizures induced by kainate injection increases transmission between the dentate gyrus and the CA3 region. This observation was validated and expanded in vitro, where the authors showed that seizure-like repetitive stimulation potentiated both the monosynaptic EPSC and the disynaptic IPSC. Despite these changes, the balance of synaptic excitation and inhibition was maintained following long-term potentiation. The authors suggest that this retained balance of excitation and inhibition might contribute to protect animals from spontaneous recurrent seizures.

These are interesting findings that deserve publication. The manuscript is well written and easy to follow. Below are concerns which dampened my enthusiasm for this manuscript.

1) I am puzzled by the size of the putative MF EPSC generated in CA3 pyramidal cells for in vitro experiments. The authors find an average amplitude of 12 pA for MF EPSC, which is at least an order of magnitude below the values reported by many laboratories (including Jonas, Castillo, McBain, Tóth, Contractor, Nicoll, Mulle, among others). Most studies at the MF-CA3 synapse report EPSCs in the range of 100 - 300 pA, especially for an external solution containing elevated Ca2+ (2.4 mM reported here). Despite the authors’ claim that the input was DCG-IV sensitive and demonstrated facilitation upon repetitive stimulation, the amplitude of EPSCs casts doubts on the MF origin of the EPSCs recorded during in vitro experiments.

2) What is the origin of the increased IPSC? Is it plasticity at the MF->interneuron synapse or interneuron->CA3 pyramidal cell synapse? Maybe it’s both. The authors should record directly from interneurons to answer this question.
